# Papular lesions on the forearms in a 56-year-old woman

**DOI:** 10.1016/j.jdcr.2025.08.037

**Published:** 2025-09-18

**Authors:** Samet Öner, Ceylan Avcı, Banu Lebe

**Affiliations:** a Department of Dermatology, Dokuz Eylül University Hospital, İzmir, Türkiye; b Department of Pathology, Dokuz Eylül University Hospital, İzmir, Türkiye

**Keywords:** acral persistent papular mucinosis, cutaneous mucinoses, dermatopathology, localized lichen myxedematosus, mucinous dermatosis

## Case description

**Fig 1:**
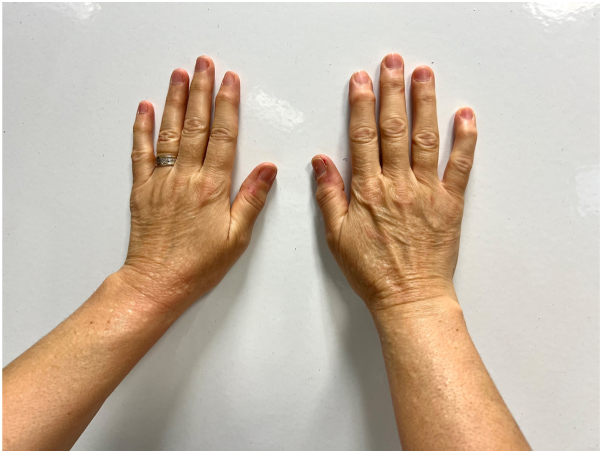
Bilateral dorsal hands and distal forearms with numerous 2–5 mm, firm, dome-shaped, flesh-colored papules in a symmetric acral distribution.

**Fig 2:**
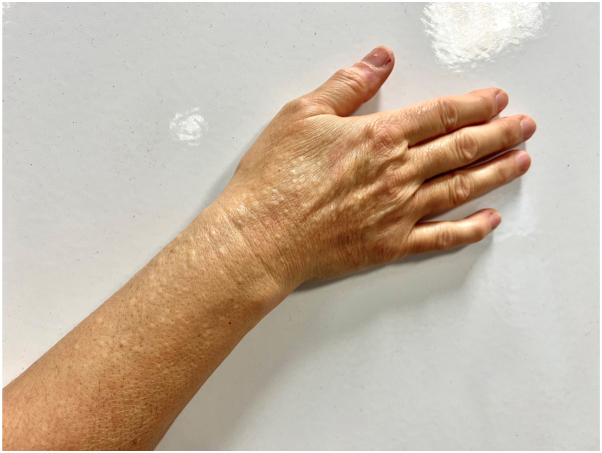
Right hand and distal forearm showing clustered, skin-colored papules over the wrist and extensor surface without erythema or scale.

**Fig 3:**
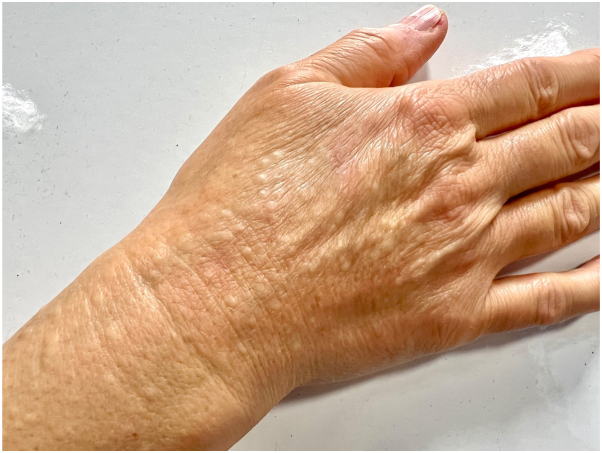
Right dorsal hand, close-up view demonstrating discrete, flesh-colored papules separated by normal intervening skin; no inflammation or secondary change.

**Fig 4:**
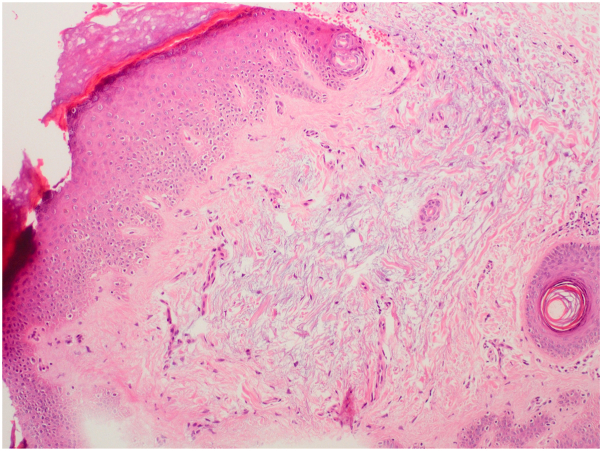
Hematoxylin–eosin (H&E) stain showing basophilic mucinous degeneration between dermal collagen bundles; original magnification ×10.

A 56-year-old woman presented with a 4-y history of persistent, asymptomatic papules symmetrically located on the dorsum of her hands and distal forearms (Fig 1). There was no history of trauma, systemic illness, or medication use. Her personal and family history were noncontributory. Dermatologic examination revealed numerous 2-5 mm, firm, dome-shaped, flesh-colored papules without inflammation or secondary changes (Figs 2 and 3). A punch biopsy demonstrated orthokeratotic epidermis with prominent mucinous degeneration in the superficial dermis separating collagen bundles without fibroblast proliferation (Fig 4). Histochemical staining with Alcian blue was positive for mucin. Special stains, including Congo red, crystal violet, PAS, and elastic tissue stain, were negative for amyloid, fungi, and elastic fiber alterations.

**Question: Which of the following is the most likely diagnosis?**

A. Papular granuloma annulare
B. Discrete papular lichen myxoedematosus
C. Acral persistent papular mucinosis
D. Eruptive xanthomas
E. Papular sarcoidosis

## Answer and Discussion

**C.** Correct answer: Acral persistent papular mucinosis. Acral persistent papular mucinosis (APPM) is a rare subtype of localized lichen myxedematosus that presents with symmetrical, flesh-colored papules on acral regions, especially the dorsum of the hands and forearms. It is histologically characterized by mucin deposition within the upper and mid-dermis, with preserved collagen and elastic fibers.^1^, ^2^, ^3^

APPM is distinguished from scleromyxedema by the absence of systemic involvement, monoclonal gammopathy, and facial distribution. Histopathologically, it typically lacks fibroblast proliferation and dermal fibrosis, features that are observed in other variants of lichen myxedematosus.^1^^,^^3^ Clinically, while discrete papular lichen myxedematosus manifests with scattered papules on the trunk and proximal extremities, APPM is characteristically confined to acral sites.^1^^,^^2^

Special stains such as Alcian blue highlight mucin, while Congo red and crystal violet are useful for excluding amyloid deposition. Serologic tests for HIV and hepatitis C virus were negative, and both TSH and free T4 levels were within normal limits. These assessments were performed due to reported associations between localized variants of lichen myxedematosus and systemic conditions such as viral infections and thyroid dysfunction.^1^^,^^2^

In cases with a classic clinical and histopathologic presentation of APPM, screening for systemic gammopathy with serum protein electrophoresis and immunofixation electrophoresis may not be necessary. However, if there are atypical features or any systemic symptoms suggestive of scleromyxedema, these tests should be considered to rule out an underlying monoclonal gammopathy.^1^^,^^3^

Although treatment is generally not required for APPM due to its benign and asymptomatic nature, some patients may seek therapy for cosmetic reasons. In our case, a 1-month trial of topical clobetasol propionate was ineffective, and the patient opted to discontinue treatment due to minimal cosmetic concern. This aligns with previous reports indicating limited efficacy of topical corticosteroids in APPM. Other options, such as tacrolimus or intralesional steroids, have shown inconsistent results.^2^^,^^3^ Given the indolent nature of APPM, treatment should be tailored to patient preference and clinical presentation.

## Conflicts of interest

None disclosed.
